# Zinc Oxide Coated Tin Oxide Nanofibers for Improved Selective Acetone Sensing

**DOI:** 10.3390/nano8070509

**Published:** 2018-07-09

**Authors:** Haiying Du, Xiaogan Li, Pengjun Yao, Jing Wang, Yanhui Sun, Liang Dong

**Affiliations:** 1 Key Laboratory of Microelectronic Devices & Integrated Technology, Institute of Microelectronics, Chinese Academy of Sciences, Beijing 100029, China; duhaiying@dlnu.edu.cn; 2 College of mechanical and Electronic Engineering, Dalian Minzu University, Dalian 116600, China; syh@dlnu.edu.cn; 3 School of Electronic Science and Technology, Dalian University of Technology, Dalian 116023, China; 4 Department of Electrical and Computer Engineering, Iowa State University, Ames, IA 50011, USA; ldong@iastate.edu; 5 School of Educational Technology, Shenyang Normal University, Shenyang 110034, China; yaopj@synu.edu.cn

**Keywords:** electrospinning, 3D hetero-nanofibers, heterojunctions, gas sensors, gas-sensing mechanism

## Abstract

Three-dimensional hierarchical SnO_2_/ZnO hetero-nanofibers were fabricated by the electrospinning method followed with a low-temperature water bath treatment. These hierarchical hollow SnO_2_ nanofibers were assembled by the SnO_2_ nanoparticles through the electrospinning process and then the ZnO nanorods were grown vertically on the surface of SnO_2_ nanoparticles, forming the 3D nanostructure. The synthesized hollow SnO_2_/ZnO heterojunctions nanofibers were further employed to be a gas-sensing material for detection of volatile organic compound (VOC) species such as acetone vapor, which is proposed as a gas biomarker for diabetes. It shows that the heterojunction nanofibers-based sensor exhibited excellent sensing properties to acetone vapor. The sensor shows a good selectivity to acetone in the interfering gases of ethanol, ammonia, formaldehyde, toluene, and methanol. The enhanced sensing performance may be due to the fact that *n*-*n* 3D heterojunctions, existing at the interface between ZnO nanorods and SnO_2_ particles in the SnO_2_/ZnO nanocomposites, could prompt significant changes in potential barrier height when exposed to acetone vapor, and gas-sensing mechanisms were analyzed and explained by Schottky barrier changes in SnO_2_/ZnO 3D hetero-nanofibers.

## 1. Introduction

Volatile organic compounds (VOCs) exist in the earth’s atmosphere from a variety of sources, which are closely related to breathing and the living environment of human beings. The detection and monitoring of VOC gas is very important due to environmental pollution. The detecting techniques and respective approaches for VOCs still require improvement when applied in the area of medicine and the environment. Metal oxide semiconductor sensors are widely used in the detection of VOCs due to rapid response, high sensitivity, good stability, small size, and simple operation. In order to improve the semiconductor gas sensor for the VOCs recognition and response, researchers are devoted to improving the preparation method for metal oxides. Electrospinning is a simple and effective method to prepare nanofibers [1]. A variety of electrospun nanofibers exhibit interesting physical and chemical characteristics and provide a large specific surface area, suitable porosity, fine fibrous structure, high mechanical flexibility, and strong maneuverability [2,3,4]. Electrospinning technology has greatly expanded its ability from preparation of organic polymeric nanofibers [2,5,6] to synthesis of various inorganic and semiconductor-based nanofibers, such as SnO_2_ [7], In_2_O_3_ [8], TiO_2_ [9], WO_3_ [10], ZnO [11], NiO [12], BaTiO_3_ [13,14], and so on.

Single metal oxide materials are unable to meet increasing requirements of material properties for a wide range of applications, especially for detection of gaseous species. Addition of the second component as a surface modifier alters electrical conductance of oxide films [15,16]. Composite materials, including both metal oxide and surface modifiers, have been proven an effective approach to improve physical and chemical characteristics of gas-sensing electrospun nanofibers. For instance, Liu, Z. et al. [17] fabricated TiO_2_/SnO_2_ nanofibers with controllable heterojunctions by electrospinning with a side-by-side dual spinneret, and TiO_2_/SnO_2_ showed a high photocatalytic activity. NiO-doped SnO_2_ nanofibers synthesized via electrospinning provided good formaldehyde-sensing properties at an operating temperature of 200 °C [18]. The heterostructure of Li-rich/Co_3_O_4_ nanoplates was reported to enhance the electrochemical performance and the electrochemically active Li*x*CoO*y* [19]. Semiconductor heterostructure can also enhance gas-sensing properties with an enhanced thermal stability [20,21].

This paper reports the development of electrospun SnO_2_/ZnO 3D hetero-nanofibers for detection of VOCs. Briefly, the SnO_2_/ZnO fibrous nanocomposite was formed by synthesizing SnO_2_ nanofibers via electrospinning and growing ZnO nanorods on the nanofiber surface in a low-temperature water bath. The morphological, structural, and composition properties of the obtained electrospun SnO_2_/ZnO 3D hetero-nanofibers were characterized and analyzed. In addition, an indirectly heated gas sensor was built with the SnO_2_/ZnO nanofibers and tested in the presence of low-concentration acetone. The SnO_2_/ZnO sensor shows excellent acetone-sensitive properties due to the existing n–n heterojunction in the electrospun SnO_2_/ZnO 3D nanocomposite. The gas-sensing mechanism of the SnO_2_/ZnO sensor was analyzed and explained by energy band changes before and after equilibration caused by the heterojunction in SnO_2_/ZnO 3D hetero-nanofibers. Finally, the electrical characterization of the Schottky diode was certificated by IV characteristics of the SnO_2_/ZnO 3D hetero-nanofibers sensor.

## 2. Experiment and Characteristics

### 2.1. Materials

Stannous chloride dihydrate (SnCl_2_·2H_2_O) and Zinc nitrate hexa-hydrate (Zn(NO_3_)_2_·6H_2_O) were purchased from Tianjin Ker-mel Chemical Corporation, Tianjin, China. Polyvinyl pyrrolidone (PVP, Mw = 1,300,000 g/mol) was purchased from Sigma-Aldrich, LS, USA. N,N-Dimethylformamide (DMF) and ethanol (EtOH) were obtained from Sinopharm Chemical Reagent Co., Ltd., Shanghai, China. The above chemical reagents are analytical grades without further purification.

### 2.2. Electrospinning of SnO_2_ Nanofibers

1.2 g SnCl_2_·2H_2_O were dissolved in 8 ml ethanol under vigorous stirring for 1 h. Subsequently, 1.2 g PVP and 6 ml DMF were added into the as-prepared SnCl_2_ solution under vigorous stirring until the PVP and DMF were thoroughly dissolved in the SnCl_2_ solution.

The prepared precursor solution was loaded into the syringe of a conventional electrospinning setup. The high D.C. voltage of 20 kV was applied at the metallic spinneret. The fiber collector was connected to the ground. The distance between the spinneret and the collector was 15 cm. The inside diameter (ID) of the spinneret was 0.41 mm. The liquid jet was ejected from the spinneret to the collector for further thermal annealing treatment at 600 °C for 2 h in the air. Finally, the SnO_2_ nanofibers were obtained.

### 2.3. Synthesis of ZnO Nanorods

0.5 g SnO_2_ nanofibers were mixed with deionized water to form a paste. The paste was coated onto a glass slide with the dimensions of 5 cm × 1 cm. Subsequently, the coated substrate was placed in an oven at 200 °C for 30 min. Meanwhile, an ethanol solution of zinc acetate (Zn(CH_3_COO)_2_∙2H_2_O) with a concentration of 0.05 mol∙L^−1^ was ultrasonically agitated for 30 min. The obtained zinc acetate solution was dripped onto the glass slide coated with SnO_2_ nanofibers. After that, the coated glass slide was dried in the oven at 200 °C for 30 min to form ZnO seeds on the surfaces of SnO_2_ nanofibers. Following that, the substrate was placed in the zinc acetate solution with a concentration of 0.04 mol∙L^−1^ at 90 °C for 4 h. Finally, the substrate was taken out and dried in a nitrogen stream at room temperature until white SnO_2_/ZnO hetero-nanofiber powders were obtained. A schematic diagram of the process flow for fabricating SnO_2_/ZnO 3D hetero-nanofibers is shown in the supplementary material. As a comparison, ZnO nanorods were prepared on the indirectly heated sensor using a low-temperature water bath. The above obtained zinc acetate solution was dripped onto the glass slide. After that, the coated glass slide was dried in the oven at 200 °C for 30 min to form ZnO seeds. The substrate with ZnO seeds was placed in the zinc acetate solution with a concentration of 0.04 mol∙L^−1^ at 90 °C for 4 h. Finally, ZnO nanorods were obtained after being dried in a nitrogen stream at room temperature.

### 2.4. Characterization of SnO_2_/ZnO 3D Hetero-Nanofibers

The morphology, structure, and composition of the prepared SnO_2_/ZnO 3D hetero-nanofibers were characterized and analyzed by an X-ray diffraction instrument (XRD, D/Max 2400, Rigaku, Tokyo, Japan) in a 2θ region of 20–80° with Cu Kα1 radiation, field emission scanning electron microscope (FESEM, Hitachi S-4800, Tokyo, Japan), selected area electron diffraction (SAED), and transmission electron microscope (TEM, Tecnai 20, FEI Company, Hillsboro, OR, USA). The composition and contents of the nanofibers were analyzed by Energy Dispersive X-Ray Spectroscopy (EDS, Tecnai 20, FEI Company, Hillsboro, OR, USA and X-Ray photoelectron spectroscopy (XPS, ESCALAB 250Xi, Thermo Waltham, MA, USA). Finally, electrochemical properties of SnO_2_/ZnO 3D hetero-nanofibers were characterized and analyzed by a semiconductor device parameter analyzer (Agilent B1500A, Agilent, Santa Clara, CA, USA).

### 2.5. Fabrication and Testing of Gas Sensors

An indirectly heated gas sensor was designed and fabricated with the prepared SnO_2_/ZnO 3D hetero-nanofibers [18]. To form the sensor, the as-prepared SnO_2_/ZnO powders were first ground with deionized water in an agate mortar to form a paste. The SnO_2_/ZnO paste was then coated onto the surface of a ceramic tube to form a sensitive film with 100~200 µm thickness, on which a pair of parallel gold electrodes was pre-plated and two pairs of platinum wires were led from each gold electrode, respectively. A Ni–Cr heating wire was inserted into the ceramic tube [22].

The sensor was measured using a static state gas-sensing test system [20]. A certain amount of target gas (*V*) was taken out from the gas cylinder with known concentration (*v*%) by a gas syringe and then injected into a 50-L sealed static state testing chamber. For a required measured concentration, the calculation formula of injected target gas volume (*V*) is as follows:

(1) $$V=\frac{50\times C}{v\%}$$

where *C* is the concentration of the target gas (ppm) to be measured and *v*% is the volume fraction of the bottled gas with known concentration. The sensor response (*S*) was defined as a ratio of the stable resistance of the sensor in the air (*R*_a_) to that in target gas (*R*_g_): *S* = *R*_a_/*R*_g_.

## 3. Results and Discussion

### 3.1. Structural Properties

**Figure 1:**
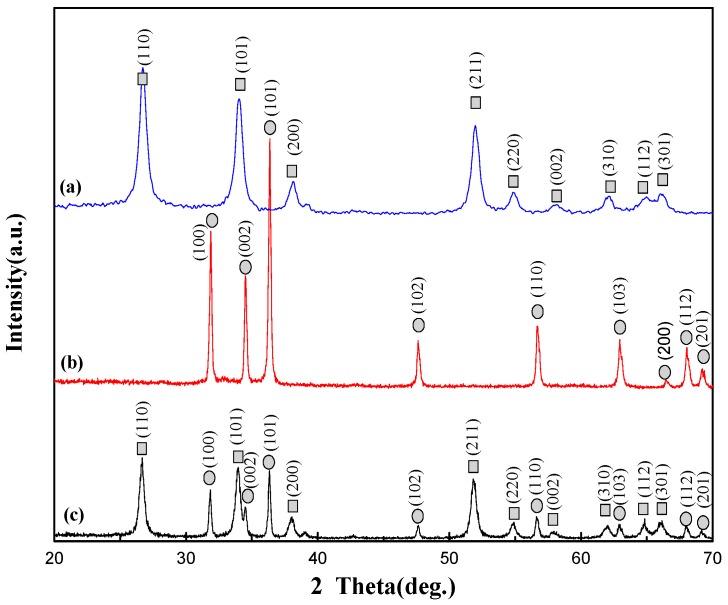
XRD patterns of (**a**) SnO_2_ nanofibers, (**b**) ZnO nanorods, and (**c**) SnO_2_/ZnO nanofibers.

The XRD patterns of SnO_2_ nanofibers synthesized by electrospinning, ZnO nanorods prepared by the low-temperature water bath method, and the final electrospun SnO_2_/ZnO 3D hetero-nanofibers are shown in Figure 1. Figure 1a,b shows that the pure SnO_2_ nanofibers belong to the rutile structure of the tetragonal phase (the square symbol), while the pure ZnO nanorods belong to the Wurtzite structure of the hexagonal phase (the circle symbol). Figure 1c shows that the characteristic peaks of both SnO_2_ and ZnO appear, indicating that the rutile structure of tetragonal phase SnO_2_ and the Wurtzite structure of hexagonal phase ZnO coexist in the prepared SnO_2_/ZnO hetero-nanofibers.

**Figure 2:**
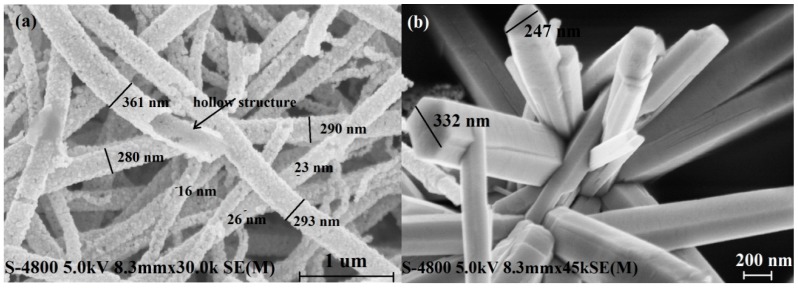

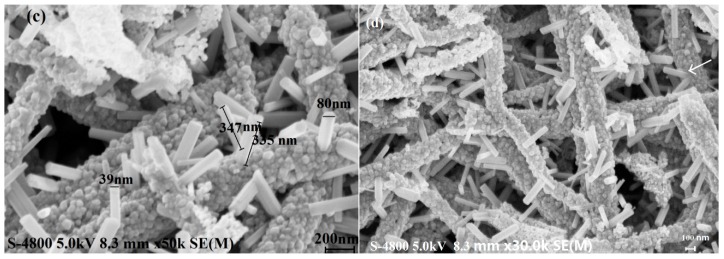
SEM images of (**a**) SnO_2_ nanofibers, (**b**) ZnO nanorods, (**c**,**d**) SnO_2_/ZnO nanofibers.

Figure 2 shows the scanning electron microscopy (SEM) images of the as-synthesized SnO_2_ nanofibers, ZnO nanorods, and SnO_2_/ZnO nanofibers. Specifically, Figure 2a demonstrates that the prepared electrospun SnO_2_ nanofibers have a hollow hierarchical structure and each nanofiber is composed of well-arranged small nanoparticles. The SnO_2_ nanofibers are also relatively uniform with a diameter of around 300 nm. Note that the diameter of SnO_2_ nanoparticles in the nanofibers is around 22 nm. Figure 2b indicates that the synthesized pure ZnO nanorods are 800~900 nm long and their cross-section has a regular hexagonal shape with a side length of 100~300 nm. In addition, a bundle of ZnO nanorods is grown together to form a flower-like ZnO nanoball [23]. Figure 2c,d display the SEM images of the final SnO_2_/ZnO nanofibers, where each ZnO nanorod seems to be growing on the surface of a single SnO_2_ nanoparticle (functioning as a seed material). The size of ZnO nanorods grown on the surface of SnO_2_ nanofibers have decreased to 300~400 nm long, and they have a hexagonal shape with a side length of 40~80 nm, which is distributed over the surface of the SnO_2_ nanofibers in a relatively uniform manner. The roots of ZnO nanorods are well attached to the surface of the SnO_2_ nanofibers. The formed SnO_2_/ZnO 3D nanocomposite maintains its hierarchical hollow structure.

**Figure 3:**
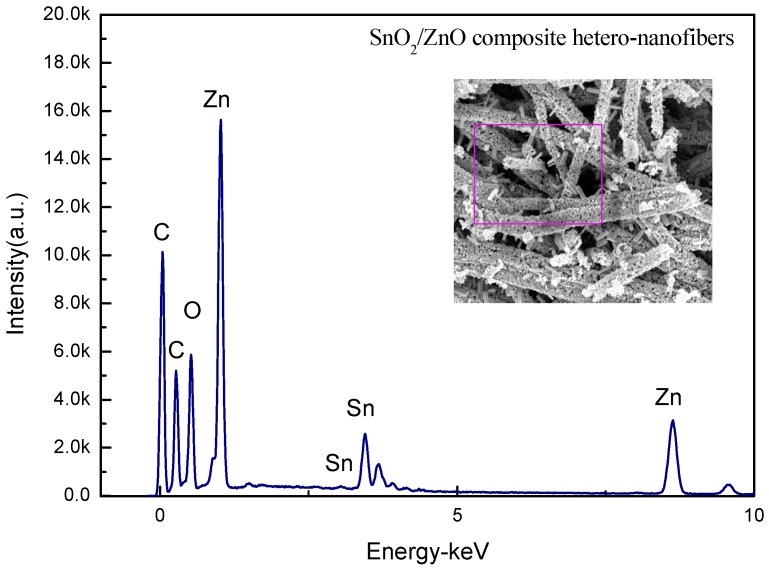
A typical EDS spectrum of the prepared electrospun SnO_2_/ZnO 3D hetero-nanofibers.

The elemental composition of the fabricated electrospun SnO_2_/ZnO 3D hetero-nanofibers was investigated using EDS. The typical EDS spectrum of this nanocomposite is given in Figure 3, where Sn, Zn, O, and C elements are present. C element is the main component of the conduction resin used in the experiment.

Table 1 lists the elemental contents of Sn and Zn in the SnO2/ZnO hetero-nanofibers. The weight percentages of Zn and Sn elements are 34.8% and 65.2%, respectively. The atomic percentages of Zn and Sn elements are 49.2% and 50.8%, respectively. It can be deduced that the atomic percentages of Zn and Sn are about 1.16, thus the ZnO and SnO_2_ present in the nanocomposite are close to equal mole quantity.

**Figure 4:**
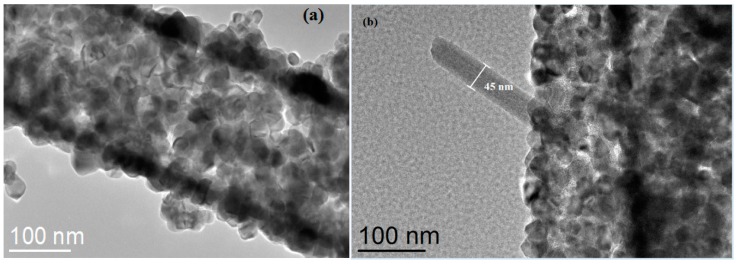
TEM images of (**a**) electrospun SnO_2_ nanofibers and (**b**) SnO_2_/ZnO 3D hetero-nanofibers.

Figure 4 shows the TEM images of electrospun SnO_2_ nanofibers and SnO_2_/ZnO 3D hetero-nanofibers. The diameters of SnO_2_ nanofibers and SnO_2_ nanoparticles shown in Figure 4a are found to be approximately 250 nm and 25 nm, respectively, which are consistent with the results of the SEM analysis shown in Figure 2a. Figure 4b confirms that the ZnO nanorod is formed on the surface of the electrospun SnO_2_ nanofiber. Hence, the SnO_2_/ZnO 3D hetero-nanofibers are synthesized with a hierarchical hollow structure.

**Figure 5:**
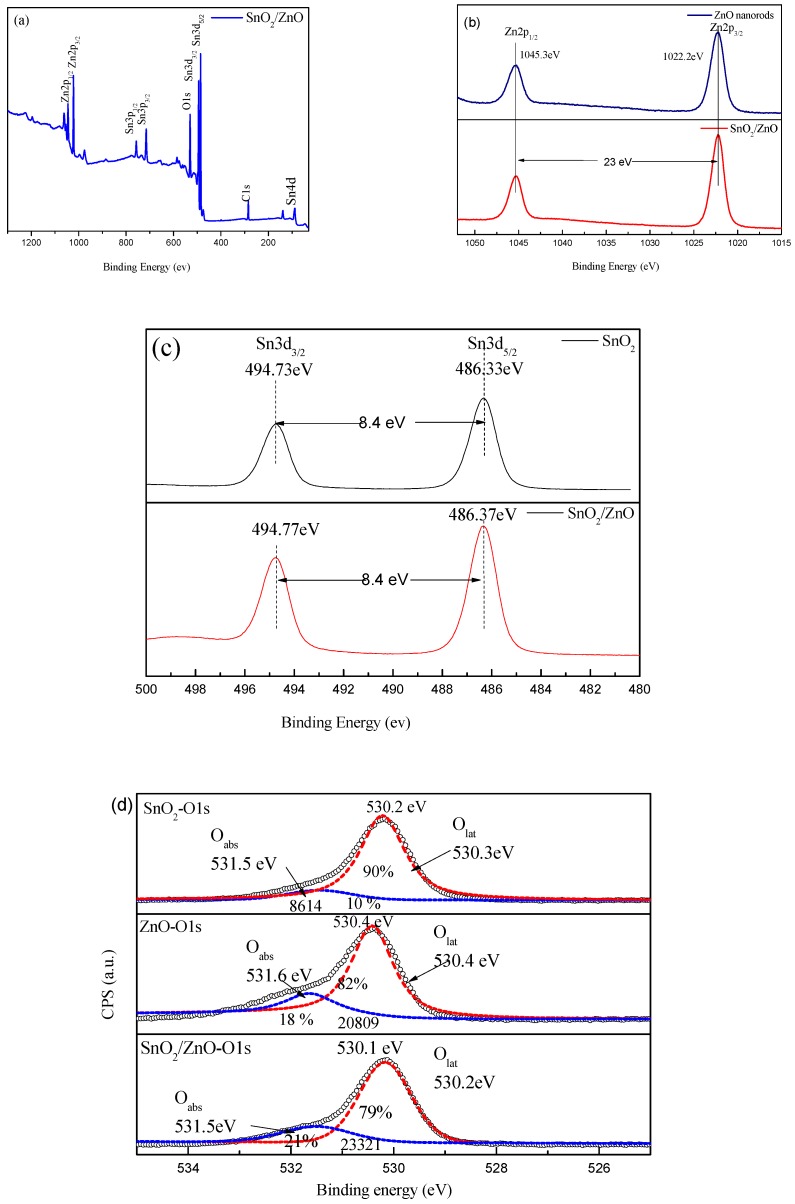
XPS spectra of SnO_2_/ZnO 3D hetero-nanofibers. (**a**) XPS spectra of SnO_2_/ZnO hetero-nanofibers. (**b**) XPS spectra of the Zn2p in ZnO and SnO_2_/ZnO. (**c**) XPS spectra of the Sn3d in SnO_2_ and SnO_2_/ZnO. (**d**) XPS spectra of the O1s in the SnO_2_, ZnO, and SnO_2_/ZnO 3D hetero-nanofibers.

XPS studies were conducted to analyze the composition and chemical states of the elements in the fabricated SnO_2_/ZnO nanofibers. The binding energy was calibrated internally by the C 1s line position. All the binding energy values were referenced to the C 1s photoemission line at 284.6 eV. Figure 5a shows nine main characteristic peaks of both SnO and ZnO components in the SnO_2_/ZnO nanofibers, including Zn2p_1/2_(1045.0), Zn2p_3/2_(1022.0), Sn3p_1/2_ (757.0 eV), Sn3p_3/2_ (715.0 eV), O1s (530.0 eV), Sn3d_3/2_ (494.0 eV), Sn3d_5/2_ (486.0 eV), and Sn4d (88.0 eV). The trace amounts of C1s (284.0 eV) may be due to the absorption of organic molecules in the air. The XPS spectra of Sn3d, Zn2p, and O1s determine the surface electronic states of Sn, Zn, and O in the nanocomposite. The binding energy of 23 eV exists between Zn2p_1/2_ and Zn2p_3/2_ peaks, and 8.4 eV between Sn3d_3/2_ and Sn3d_5/2_, as shown in Figure 5b,c, respectively. In comparison, the XPS spectra of Sn3d of SnO_2_ and Zn2p of ZnO are measured and shown in Figure 5b,c. The O1s core level XPS spectra of the SnO_2_, ZnO, and SnO_2_/ZnO nanofibers are shown in Figure 5d. O1s spectra of SnO_2_/ZnO is an asymmetric peak at 530.2 eV, which can be separated into two peaks at O_lat_ (530.1 eV) and O_abs_ (531.5 eV). The O_lat_ peak is attributed to the lattice oxygen on the surface of SnO_2_/ZnO and the O_abs_ peak is associated with the adsorbed oxygen of SnO_2_/ZnO_2_. The relative intensities of these two factors are 79% and 21%, respectively. We can see that O1s spectra of SnO_2_ are asymmetric peaks at 530.2 eV, which can be separated into two peaks at O_lat_ (530.3 eV) and O_abs_ (531.5 eV). The relative intensities of these two factors are 90% and 10%, respectively. The content of adsorbed oxygen (O_abs_) of SnO_2_ is the least of three materials. The O1s asymmetric peaks at 530.2 eV of ZnO nanorods can be separated into two peaks at O_lat_ (530.4 eV) and O_abs_ (531.6 eV). The relative intensities of these two factors are 82% and 18%, respectively. The content of adsorbed oxygen (O_abs_) of ZnO is less than that of SnO_2_/ZnO_2_, which can be seen from Figure 5d. The content of absorbed oxygen dominates the adsorption capacity of the nanomaterials, which shows that the adsorption capacity of SnO_2_/ZnO_2_ is stronger than that of SnO_2_ and ZnO.

### 3.2. Gas-Sensing Properties

**Figure 6:**
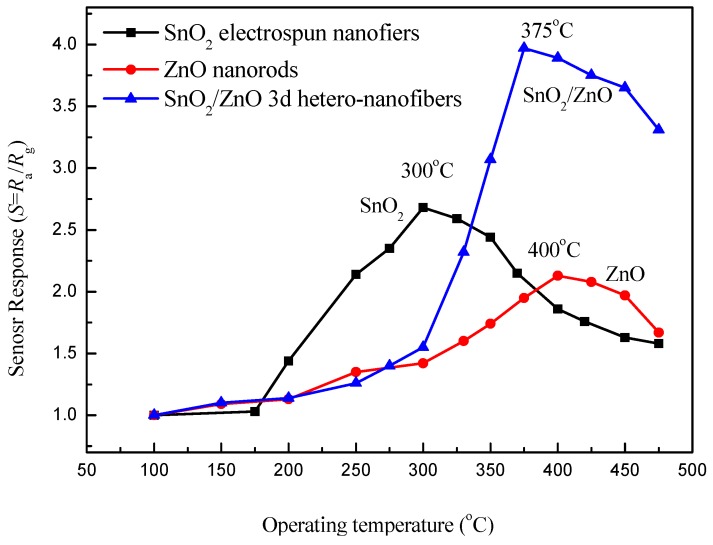
Responses of the SnO_2_, ZnO, and SnO_2_/ZnO sensors to 10 ppm concentration of acetone with 40% relative humidity under different operating temperatures.

Operating temperature is an important character of gas sensors. Figure 6 provides the responses of the sensors with SnO_2_, ZnO, and SnO_2_/ZnO to 10 ppm concentration of acetone with 40% relative humidity (RH) under different operating temperatures. The results show that the response of the SnO_2_/ZnO sensor reaches the highest value of 3.97 at 375 °C, while the response of the SnO_2_ and ZnO sensors reaches the highest value of 2.68 and 2.13 at 300 °C and 400 °C, respectively. We can see that the response of the SnO_2_/ZnO nanocomposite sensor has been improved greatly for the composite hetero-structure compared with the responses of the SnO_2_ and ZnO sensors at 375 °C. Therefore, 375 °C was chosen as the optimal operating temperature of the sensor.

**Figure 7:**
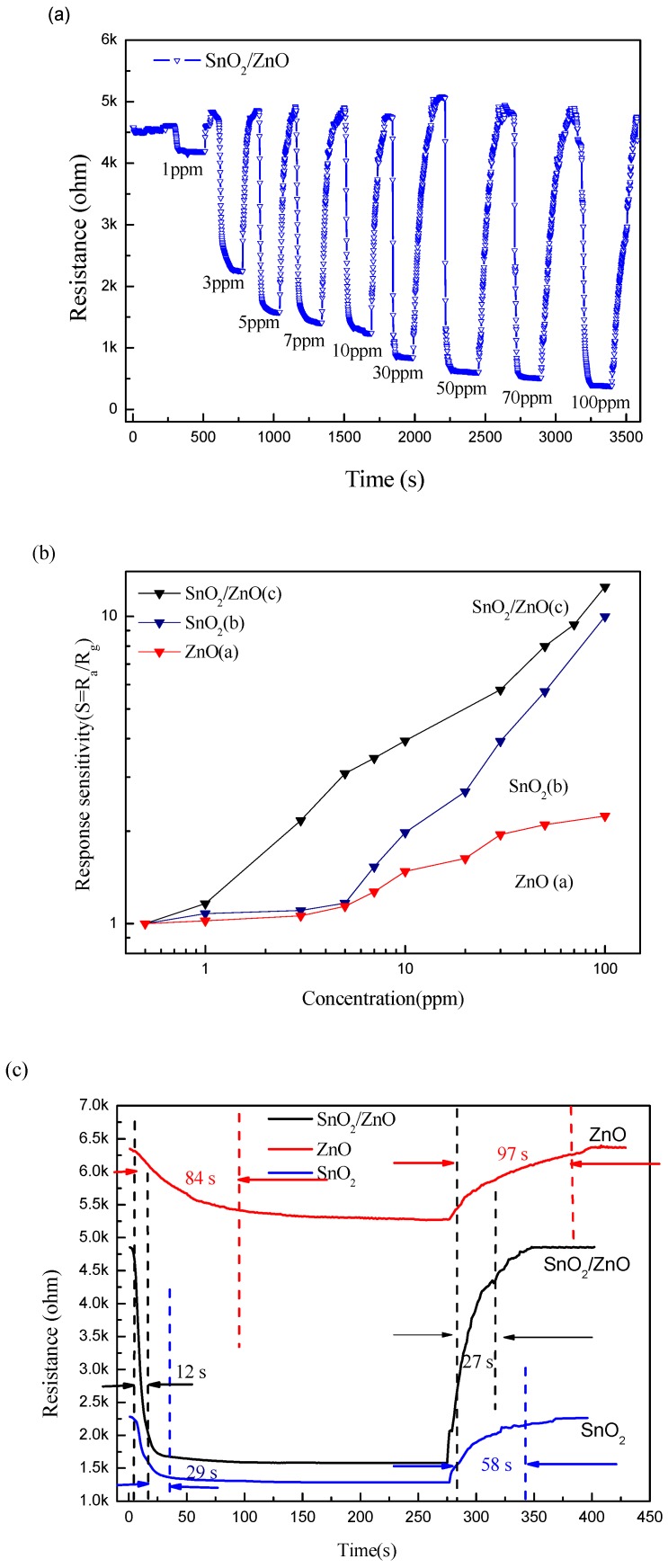
Sensing performance of the SnO_2_/ZnO sensor response to acetone. (**a**) Transient responses of the SnO_2_ /ZnO sensor to different acetone concentrations. (**b**) Response values of the SnO_2_, ZnO, and SnO_2_/ZnO sensors as a function of acetone concentration. (**c**) The response and recovery times of the SnO_2_, ZnO, and SnO_2_/ZnO sensors to 5 ppm acetone.

Figure 7a shows the transient responses of the sensor with SnO_2_/ZnO to different acetone concentrations ranging from 1 ppm to 100 ppm. The resistance change cycles of the SnO_2_/ZnO sensor were successively recorded. As the acetone concentration increases, the resistance of the sensor decreases. For comparison, Figure 7b includes the responses of the SnO_2_ and the ZnO counterpart sensors, along with the SnO_2_/ZnO sensor, as a function of acetone concentration (1~100 ppm). The SnO_2_/ZnO sensor exhibits the highest response to each concentration, compared to the sensors with SnO_2_ alone and ZnO alone. More specifically, the sensors with SnO_2_/ZnO, SnO_2_, and ZnO have the responses of 1.16, 1.08, and 1.02, respectively, when exposed to 1 ppm acetone; the SnO_2_/ZnO, SnO_2_, and ZnO sensors have the responses of 3.08, 1.17, and 1.14, respectively, to 5 ppm acetone; and three sensors have responses of 3.94, 1.98, and 1.48 to 10 ppm acetone, respectively. We can see from measurement dates of low concentration acetone that the SnO_2_ and ZnO sensors have no response basically to low-concentration acetone, while the SnO_2_/ZnO sensor has an obvious response to acetone of low concentration. Figure 7c shows that the SnO_2_/ZnO sensor has a 12-s response time and 27-s recovery time in response to acetone of 5 ppm. The response time and recovery time of the SnO_2_/ZnO sensor are much faster than those of SnO_2_ and ZnO sensors to 5 ppm acetone at 375 °C. In this experiment, the response and recovery times presented here are defined as the times required for 10% to reach 90% of the final stable values.

**Figure 8:**
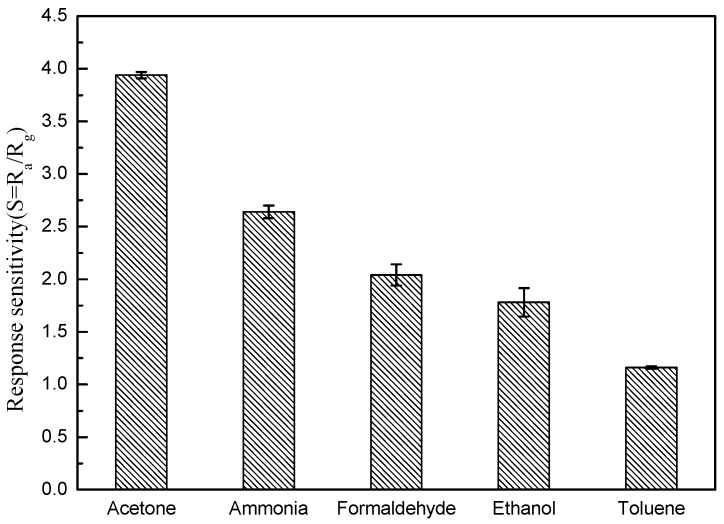
Cross-responses of the SnO_2_/ZnO sensor to acetone, ammonia, formaldehyde, ethanol, and toluene, each at 10 ppm concentration.

Figure 8 shows the cross-responses of the SnO_2_/ZnO sensor to different gas species, including acetone, ammonia, formaldehyde, ethanol, and toluene at 10 ppm. The result shows that among the five gas species tested here, the SnO_2_/ZnO sensor is the most sensitive to acetone (*S* = *R*_a_/*R*_g_ = 3.94 at 10 ppm). 

**Figure 9:**
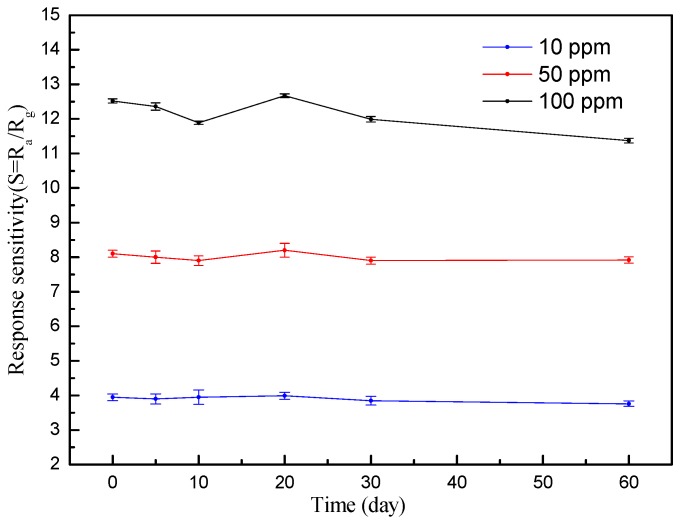
Long-term stability of the SnO_2_/ZnO sensor tested over two months.

Long-term stability and repeatability of the SnO_2_/ZnO sensor were studied over a two-month period. Figure 9 shows that the SnO_2_/ZnO sensor exhibits a relative standard deviation of 3.9% from its initial response when exposed to 100 ppm acetone concentration. However, when exposed to the lower gas concentrations of 10 ppm and 50 ppm, the sensor provided almost constant responses over two months with the RSD of 2.1% and 1.5%, respectively [24].

The test results show that the SnO_2_/ZnO sensor exhibits excellent gas-sensing properties. Recently, many researchers synthesized the SnO_2_/ZnO composites. Table 2 lists the preparation methods of SnO_2_/ZnO composites and their gas-sensing properties. We can see from Table 2 that the SnO_2_/ZnO 3D hetero-nanofibers sensor has excellent acetone-sensitive properties compared to SnO_2_/ZnO sensors reported by journals. 

## 4. Gas-Sensing Mechanism

The results demonstrate that the SnO_2_/ZnO sensor exhibits a higher response to acetone than the SnO_2_ sensor and the ZnO sensor, indicating that the adsorption capability of SnO_2_/ZnO nanofibers to acetone is greatly enhanced. The gas-sensing mechanism of the SnO_2_/ZnO sensor is explained below.

**Figure 10:**
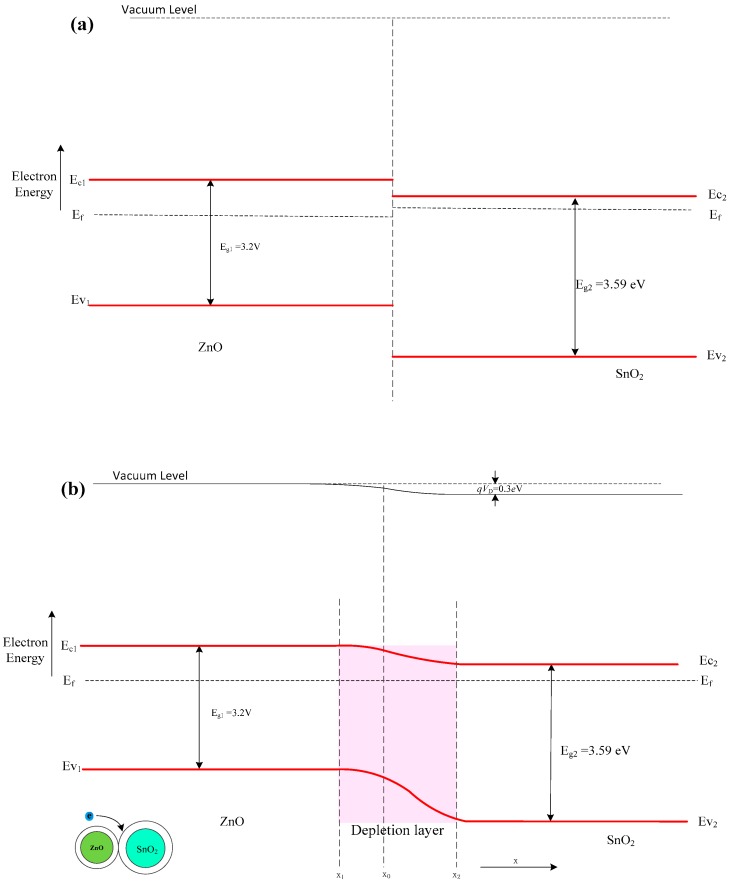
Energy band diagram of the electrospun SnO_2_/ZnO 3D hetero-nanofibers system. (**a**) Energy band diagram of SnO_2_/ZnO 3D hetero-nanofibers system before equilibrium. (**b**) Energy band diagram of SnO_2_/ZnO 3D hetero-nanofibers system at equilibrium.

At first, we try to explain using the energy band theory of semiconductors. Both ZnO and SnO_2_ are n-type, semiconductor-based, gas-sensing materials. Adsorption of oxygen (O_2_^−^, O_2_^2−^, and O^2−^ ions) on the grain surface will result in capturing electrons from the conductance band of the material to form an electron-depleted, space-charge layer in the surface region of the grain [32,33]. The electrons in the conduction band must overcome a potential barrier to move to neighboring grains. The more oxygen ions absorbed on the grain surface, the higher the potential barrier, and thus, the fewer the electrons present in the conduction band. This will increase the surface potential barrier and thus increase the resistance of the sensitive material [34,35]. The band gap and the work function of SnO_2_ are 3.59 eV and 4.9 eV, respectively, while the band gap and the work function of ZnO are 3.2 eV and 5.2 eV, respectively [36,37,38]. Thus, the Fermi energy level (E_f_) of SnO_2_ is higher than that of ZnO due to the lower work function of SnO_2_. Furthermore, SnO_2_ has a higher electron affinity (4.5 eV) than ZnO (4.3 eV); electron transfer will occur from ZnO to SnO_2_ until the energy band diagram of the *n*–*n* heterojunction of SnO_2_/ZnO come to equilibrium [39]. Because the Fermi energy level is directly related to the number of accumulated electrons, the Fermi energy of SnO_2_ and ZnO tends to shift to a higher and lower level, respectively. Thus, a new Fermi energy level will be formed in the fabricated SnO_2_/ZnO heterojunction. Figure 10a,b shows the energy band diagram of the heterojunction before and after equilibrium, respectively. Because electrons and hole transport in the heterojunction lead to energy band bending, and oxygen has a strong electronegativity, the surface activity of the SnO_2_/ZnO nanofibers becomes high enough to promote oxygen adsorption on the surface of the nanocomposite. The adsorbed oxygen can serve as traps for conduction-band electrons, causing deterioration in electrical conductivity of the SnO_2_/ZnO nanocomposite. When it meets with the reductive gas species, such as acetone, ethanol, and formaldehyde, it can absorb more oxygen from the surface of the nanocomposite. Thus, the resistance of the nanocomposite (SnO_2_/ZnO) will decrease due to desorbing oxygen. The more positive surface activity of SnO_2_/ZnO nanofibers, the smaller the resistance of the SnO_2_/ZnO sensor, and the greater the response of the SnO_2_/ZnO sensor [40,41,42].

Moreover, the 3D hetero-nanostructure will improve the porosity of gas-sensing materials. More gas channels are formed for 3D hetero-nanostructure, which make it easy and fast for target gas adsorption and desorption. The response time and recovery time of the SnO_2_/ZnO sensor are reduced greatly. The more specific surface area will provide more opportunities for the target gas to contact the surface of SnO_2_/ZnO 3D hetero-nanofibers, which will improve the gas-sensing properties of gas sensors.

**Figure 11:**
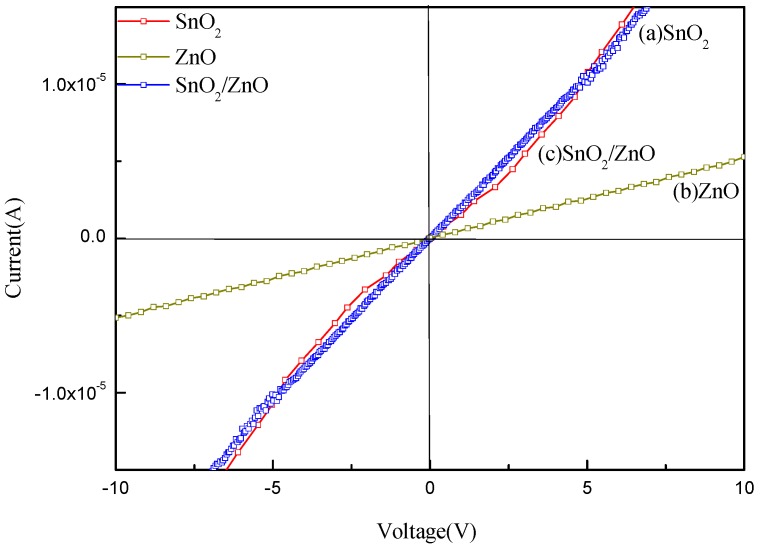
I-V curves of (**a**) the SnO_2_, (**b**) ZnO, and (**c**) SnO_2_/ZnO sensors.

In addition, the electrochemical properties of the SnO_2_, ZnO, and SnO_2_/ZnO sensors were tested at the operating temperature of 375 °C. Figure 11 shows the current-voltage (I-V) characteristics of the fabricated SnO_2_, ZnO, and SnO_2_/ZnO gas sensors. The I-V curves of the sensors with SnO_2_ nanofibers alone and ZnO nanorods alone approximate to a straight line, indicating the formation of a good ohmic contact between the sensor materials and the Au electrodes. Because ZnO has a narrower forbidden bandwidth than SnO_2_, the slope of the I-V curve for the ZnO sensor is steeper than that of the SnO_2_ sensor. The I-V curve of the SnO_2_/ZnO sensor shows nonlinearity with a rectifying property, suggesting the formation of a rectifying Schottky junction [43]. With the decreasing potential barrier of the Schottky junction, the I-V curve of the sensor exhibits a clear rectifying behavior. The electrical characterization of the Schottky diode necessitates the determination of the barrier height and the ideality factor [44]. The composites with *n*–*n* heterojunctions have a significantly different height between electron and hole barriers and their I-V curves show significant nonlinearity. While the materials with homojunctions have the same electron and hole built-in potential barriers and their I-V curves show linearity. Such heterojunctions lead to the change in the grain boundary barrier of the composites. The band of the composites is deformed, changing the transport properties of electrons so that the gas-sensing properties of nanocomposites are enhanced [45,46,47]. 

## 5. Conclusions

An electrospinning followed by a low-temperature water bath method was designed for constructing electrospun SnO_2_/ZnO 3D hetero-nanofibers. ZnO nanorods grow on the hierarchical hollow electrospun SnO_2_ nanofibers to form SnO_2_/ZnO 3D hetero-nanofibers. The gas-sensing properties of SnO_2_/ZnO 3D hetero-nanofibers sensors were tested with an acetone concentration range of 1~100 ppm. Test results showed that SnO_2_/ZnO 3D hetero-nanofibers gas sensor exhibits high response values and fast response and recovery times to acetone, and the SnO_2_/ZnO sensor shows good selectivity to acetone in the interfering gases of ethanol, ammonia, formaldehyde, toluene, and methanol. An enhanced response of SnO_2_/ZnO 3D hetero-nanofibers sensors to acetone may be due to *n*–*n* homotype heterojunctions existing in the joint between ZnO nanorods and SnO_2_ particles in the SnO_2_/ZnO nanocomposite. Heterojunctions cause the change of potential barrier height, then electronic transport properties are enhanced greatly owing to the heterojunction of SnO_2_/ZnO 3D nanocomposite, which improves the gas-sensing properties of SnO_2_/ZnO composite materials. At last, gas-sensing mechanisms of electrospun SnO_2_/ZnO 3D hetero-nanofibers were discussed and analyzed by semiconductor energy band theory.

## Figures and Tables

**Table 1:** Elemental contents of SnO_2_/ZnO 3D hetero-nanofibers.

Elements	Weight (%)	Atomic (%)
O K	16.9	56.5
Zn K	28.9	23.4
Sn L	54.2	20.1

**Table 2:** Comparison of the gas sensor based on SnO_2_/ZnO composites and their gas-sensing properties.

Types	Preparation Method	Detect Gas	Structure	Operating Temperature (°C)	Response Value (Concentration)	Response Time /Recovery Time (s)
SnO_2_/ZnO reported by other journals	Two steps electrospinning and atomic layer deposition [25]	O_2_	SnO_2_–ZnO core-shell nanofiber	300	S = 1.02 (70 ppm)	250 s/500 s
		NO_2_			S = 3.08 (5 ppm)	40 s/120 s
	A combinatorial solution deposition technique [26]	C_2_H_5_OH	SnO_2_/ZnO films	300	S = 4.69 (200 ppm)	Excellent selective
	A combination of surfactant-directed assembly and an electrospinning [21]	C_2_H_5_OH	A mesoporous structure	300	S = 4 (5 ppm)	3 s/8 s
	The pellet by sintering [27]	CO	More porous microstructure	360	S = 12 (200 ppm)	─
	The thermal evaporation of Sn powders followed by the ALD of ZnO [28].	NO_2_	SnO_2_-Core/ZnO-Shell	Room temperature	S = 1.04 (5 ppm)	110 s/230 s
	(SnO_2_) PECVD and ZnO deposited by spin coating [29].	H_2_	ZnO Surface Modifi cation of the SnO_2_ Nanorod Arrays	350	S = 2.6 (100ppm)	7 s/30 s
	Mix-electrospun [30]	CH_3_OH	Hollow hierarchical, and heterostructure	350	S = 8.5 (10 ppm)	20 s/40 s
	Two-step solvothermal method [31]	Photocatalytic Activity	Network Structured	High Photocatalytic Activity	─	─
SnO_2_/ZnO 3D hetero-nanofibers	Electrospinning followed by a low-temperature water bath treatment	Acetone	ZnO nanorod grew on the SnO_2_ nanofibers	350	S = 3.08 (5 ppm)	12 s/27 s
